# Multi-scale structural community organisation of the human genome

**DOI:** 10.1186/s12859-017-1616-x

**Published:** 2017-04-11

**Authors:** Rasha E. Boulos, Nicolas Tremblay, Alain Arneodo, Pierre Borgnat, Benjamin Audit

**Affiliations:** 1 Univ Lyon, Ens de Lyon, Univ Claude Bernard Lyon 1, CNRS, Laboratoire de Physique, F-69342, Lyon, France; 2 Present address: Montpellier Cancer Institute (ICM), Montpellier Cancer Research Institute (IRCM) Inserm U1194, University of Montpellier, Montpellier, France; 3 Present address: CNRS, GIPSA-lab, Grenoble, France; 4 Present address: LOMA, Université de Bordeaux, CNRS, UMR 5798, 51 Cours de le Libération, Talence, 33405 France

**Keywords:** Chromosome interaction network, Multi-scale community mining, Structural domain hierarchical organisation, Spectral graph wavelets, Human genome

## Abstract

**Background:**

Structural interaction frequency matrices between all genome loci are now experimentally achievable thanks to high-throughput chromosome conformation capture technologies. This ensues a new methodological challenge for computational biology which consists in objectively extracting from these data the *structural motifs* characteristic of genome organisation.

**Results:**

We deployed the fast multi-scale community mining algorithm based on spectral graph wavelets to characterise the networks of intra-chromosomal interactions in human cell lines. We observed that there exist structural domains of all sizes up to chromosome length and demonstrated that the set of structural communities forms a hierarchy of chromosome segments. Hence, at all scales, chromosome folding predominantly involves interactions between neighbouring sites rather than the formation of links between distant loci.

**Conclusions:**

Multi-scale structural decomposition of human chromosomes provides an original framework to question structural organisation and its relationship to functional regulation across the scales. By construction the proposed methodology is independent of the precise assembly of the reference genome and is thus directly applicable to genomes whose assembly is not fully determined.

**Electronic supplementary material:**

The online version of this article (doi:10.1186/s12859-017-1616-x) contains supplementary material, which is available to authorized users.

## Background

**Fig. 1:**
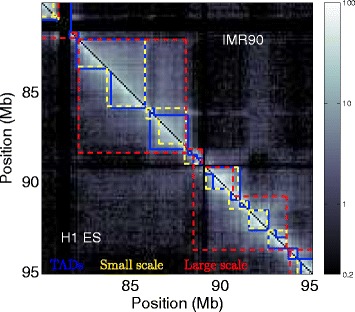
Hi-C co-localisation maps reveal a multi-scale structural organisation. Hi-C co-localisation frequency matrices along a 15 Mb fragment of human chromosome 10 in H1 ES (resp. IMR90) under (resp. above) the *diagonal* with intensity of interactions colour coded according to colour map on the right. Blue lines represent TADs [11] in the two cell lines. Coloured dashed lines correspond to 2 partitions into communities obtained at small (*yellow*) and large (*red*) scales. Columns and rows in *black* correspond to masked regions (Methods and Additional file 1: Table S1)

It is now well established that eukaryotic genome dynamics and 3D architecture have a fundamental role in the regulation of nuclear functions such as DNA replication and gene transcription [1–6]. At small scale (∼200 bp), the crystal structure of the nucleosome core particle (the first level of eukaryotic DNA compaction formed by complexing ∼150 bp of DNA with 8 histone proteins) was determined 20 years ago [7]. At the scale of the nucleus, fluorescence imaging revealed the dominant structural organisation of the genome into *chromosome territories* reflecting a non-mixing compartmentalisation of the chromosomes [2]. However, until the emergence of Chromatin Conformation Capture (3C) technologies [8, 9], our knowledge of the structural organisation of DNA at the intermediary scales remained partial. High-throughput 3C protocol (Hi-C technique) has opened new perspectives in the study of these intermediary structures genome-wide in higher eukaryotes, closing the gap between the atomic and chromosomal resolutions [10–18]. Hi-C technique relies on high-throughput sequencing and allows to semi-quantitatively measure the co-localisation frequencies of all pairs of genomic loci (the spatial resolution of the most recent data [19, 20] is ∼1−10 kb for mammalian genomes of length ∼3 Gb). Inter-chromosome co-localisation frequencies are lower than intra-chromosome frequencies, following the nuclear organisation into chromosome territories [10]. Mean intra-chromosome frequencies decrease with the genomic distance as expected for a polymer [21]. Changes in the decreasing rate reflect the modifications of the global chromosome structure like the chromosome condensation observed during entry in metaphase [19]. Nevertheless Hi-C data also put into light a structural compartmentalisation of the genome at different scales that cannot be explained by simple homogeneous polymer models [22]. Principal component analysis of the correlation matrix between the co-localisation frequency profiles of each locus revealed the existence of two nuclear compartments, loci preferentially co-localising with other loci from the same compartment: compartment A is associated with gene rich and early replicating regions and compartment B with gene poor and late replicating regions [10]. Projected on the genome, this classification describes the chromosomes as the succession of A/B domains of length ∼10 Mb. Inspection of intra-chromosomal co-localisation frequency matrices reveals a finer structuring level characterised by diagonal blocks of length ∼0.1−1 Mb: co-localisation frequency is high between regions of the same block but weaker between regions belonging to different blocks [11] (Fig. 1). These blocks, named Topologically Associating Domains (TADs), underline a structural compartmentalisation of chromosomes whose link with genome functional organisation and dynamics is the subject of intense research activity [11, 15, 16, 19, 20, 23–29]. In order to carry out this research, methods allowing to objectively delineate structural domains from Hi-C data have been developed [11, 16, 26–34]. Most of these approaches look for structural domains that are intervals of the chromosomes. For example, chromosome structural partition was achieved using (i) 1D signals quantifying the balance between the co-localisation frequencies of the locus of interest with upstream and downstream loci (directionality index) [11, 27], (ii) dynamic programming algorithms that also explicitly model structural domains as chromosome intervals [31, 32] and (iii) projecting on the genome the bisection obtained from a graph representation of the Hi-C data (see below) [28, 34]. As illustrated in Fig. 1, chromosome structural organisation can involve nested structures over a large range of scales [22, 29]. However only the method proposed in [31] explicitly includes the possibility to identify chromosome structural domains at diverse scales of observation and the method in [29] to hierarchically merge adjacent TADs into *metaTADs*.

Here we propose a novel method to analyse Hi-C data that allows a multi-scale identification of structural domains. Because it does not rely on the specific assembly of the reference genome, this method does not look for structural domains limited to chromosome intervals thereby relaxing our preconception about the nature of structural domains. Moreover, due to polymorphisms within a species or to chromosome rearrangements characteristic of cancer cells [35], the assembly of the reference genome does not necessarily corresponds to the true assembly for a cell line under investigation. In these situations, reduced sensitivity to genome assembly is likely to avoid erroneous structural domain predictions. A Hi-C co-localisation frequency matrix is positive and symmetric, it can thus be interpreted as the adjacency matrix of the genome interaction network where the nodes are the chromosome loci (typically non-overlapping windows) and the edges reflect the co-localisation frequency between these regions. This justifies the use of concepts and tools from graph theory to analyse Hi-C data [28, 30, 33, 34, 36–38]. This representation depends on genome assembly only up to the scale used to define the Hi-C matrix, the columns/rows of the Hi-C matrix can be permuted without affecting the output of graph algorithms. In graph theory, a set of nodes that share more connections between themselves than with the rest of the graph is called a *community* [39]. Hence we reformulate the question of structural domain mining as a search for community in the Hi-C interaction network. Note that Markov graph clustering was already experienced to delineate sub-segments within large A/B-like chromosomal domains obtained in a first step [30] and that extensions of graph stochastic block models were also applied to Hi-C matrices of human chromosome 4 and a segment of human chromosome 6 [33]. In order not to privilege any particular scale in the analysis, we performed the multi-scale partitioning of the full intra-chromosomal interaction networks into structural communities using a multi-scale community mining algorithm based on graph wavelets [40].

## Methods

### Chromatin conformation capture data and topologically associating domains

Here we used Hi-C data obtained in different human cell lines:

- Embryonic stem cell line H1 (H1 ES) and foetal lung fibroblast cell line IMR90 Hi-C data for which TADs are available [11], allowing a direct comparison of our structural communities with what is considered as reference structural domains in the literature. Hi-C matrices at resolution 20 kb and 40 kb for two replicates in each cell lines as well as TADs predictions in these cell lines were downloaded from the GEO database under accession number GSE35156. These data are based on the hg18 assembly version of the human genome.
- Myelogenous leukemia cell line K562 and lymphoblastoid cell line GM06990 Hi-C data [10] for the analysis of the structural conservation between cell lines. Hi-C matrices at resolution 100 kb for the two cell lines were downloaded from the GEO database under accession number GSE18199. These matrices are based on the hg18 assembly version of the human genome.
- Cervical cancer cell line HeLaS3 Hi-C data [19] where the Hi-C experiments were performed on synchronised cells during mitosis and G1 allowing a study of the community structure during the cell cycle. The Hi-C reads alignment files to the human genome (hg19 assembly version) for the two stages of the cell cycle were downloaded from the ArrayExpress database under accession number E-MTAB-1948.

Hi-C intra-chromosomal co-localisation frequency matrices for non-overlapping 100 kb loci correspond to the downloaded matrices that were down-sampled to 100 kb when necessary or were constructed from the alignment files (Fig. 1). Unexpectedly low and unexpectedly high interacting loci that are likely to introduce noise were removed (Additional file 1: Table S1). The remaining 100 kb loci were concatenated resulting in new *masked positions*.

We compared the structural-communities described in this work to the TADs [11] that are considered as a reference for the structural description of Hi-C data. TADs were identified in H1 ES and IMR90 cell lines at both 20 and 40 kb resolutions [11]. Given our adopted resolution of 100 kb, we used the TADs dataset obtained at the 40 kb resolution, and we assigned each TAD border to the corresponding 100 kb pixel keeping only TADs larger than 200 kb (3 pixels). This led to a database of 2 993 (resp. 2 263) TADs in H1 ES (resp. IMR90), with 3 905 (resp. 3 096) distincts borders in H1 ES (resp. IMR90).

In this work one focus is to question the existence of a TAD-like structuration of the human genome in the intermediary scale range from the described TAD typical size up to the chromosome length. A second objective is to address the possible conservation of these structural motifs across cell lines. This led us to include the K562 and GM06990 datasets from the original Hi-C study [10]. These datasets are less resolutive than more recent ones in IMR90 and H1 ES cell lines [11] due to a limited sequencing depth and were analysed at best at 100 kb resolution by the original authors. This explains why we chose 100 kb as the resolution for all the analysis presented in our manuscript. However to check whether lower or higher resolution has significant impact on the results, the IMR90 dataset was also analysed at resolutions 40 and 200 kb.

### Multi-scale community mining using graph wavelets

We used the multi-scale community mining algorithm based on spectral graph wavelets that we previously described and benchmarked against two other multi-scale community mining methods from the literature [40]. The purpose of detecting communities at different scales using graph wavelets instead of, say, cutting a hierarchical clustering at different levels, is to fit as close to the data as possible. Cutting a hierarchical clustering impose a hierarchical structure to the set of community obtained at the different scales (cutting levels). When using wavelets, we do not suppose beforehand that the data have a hierarchical structure: a community at a coarse scale does not necessarily have to contain communities found at a finer scale.

Our community mining algorithm [40] relies on the precise construction of graph wavelets in order to introduce the notion of scale [41] (Supplementary text: Graph wavelet transform and community mining and Figures S1 to S4 in Additional file 1). A graph wavelet centred on a node *a* is a function on the nodes of the graph whose values capture the *proximity* of each node to node *a* given a scale *s* of observation. As such, the set of graph wavelets at scale *s* characterise the local graph structure around each node over a “distance” controlled by the scale parameter *s*, as illustrated in Additional file 1: Figures S1 and S3. At a fixed scale, the similarity between the neighbourhood of 2 nodes (*a* and *b*) can be quantified as the correlation ($\mathcal {C}^{(s)}(a,b)$, Additional file 1: Equation (S11)) between the wavelets centred on each of the two nodes at that scale. Computing the correlation distance $\mathcal {D}^{(s)}(a,b)=1-\mathcal {C}^{(s)}(a,b)$ (Additional file 1: Equation (S12)) between all pairs of nodes results in a distance matrix capturing the similarity of node neighbourhood, which can in turn be used as the input of a hierarchical clustering algorithm in order to partition the nodes into communities for the scale of observation *s* (Additional file 1: Figure S4). To sum up, at each scale and for each intra-chromosomal interaction network, the community mining algorithm amounts to (i) compute the matrix of correlation distance $\mathcal {D}^{(s)}(a,b)$, (ii) apply average-linkage hierarchical clustering [42, 43], and (iii) finally cut the resulting dendrogram following the method prescribed in [40]. This results in a set of structural communities for a given scale and a given chromosome.

We used the fast implementation of this procedure [40]. On the one hand, the graph wavelet transform is computed using the fast algorithm proposed in [41] (Additional file 1: Equation (S17)). On the other hand, instead of computing the wavelets on the *n* nodes of the graph which requires *n* wavelet transforms of Dirac functions (using Additional file 1: Equation (S17) *n* times), the matrix of correlations between wavelets at scale *s* is approximated by the correlation between *η* (≪*n*) wavelet transforms of random Gaussian functions on the graph (Supplementary text: Graph wavelet transform and community mining in Additional file 1). Importantly, the fast implementation of our multi-scale community mining protocol is applicable to large networks with $\gtrsim 10\;000$ nodes [40], allowing to consider its future application to intra-chromosomal interaction networks at high resolution (∼10 kb) in mammals [20] but also to full genome interaction networks at the resolution used in the present work (100 kb). Note that graph spectral clustering can also be considered for these large interaction network settings thanks to recent algorithmic developments [44, 45].

### Comparing sets of genomic domains

As discussed in Results and discussion, communities within intra-chromosomal interaction networks can be described in terms of genomic intervals i.e. sets of loci that form contiguous genomic domains and can thus be fully described by their two extreme positions, called domain borders. We adopted the three following points of view for the comparison of sets of genomic domains (chromosome intervals) of different origins. Note that because the sets of domains of interest here do not form partitions of the genome, we could not adopt the classical measures of similarity between partitions like Mutual Information and Adjusted Rand Index. Given two sets of domains $\mathcal {D}_{1}$ and $\mathcal {D}_{2}$ with two sets of associated borders $\mathcal {B}_{1}$ and $\mathcal {B}_{2}$ respectively, we used the following estimators:

- Mean best mutual coverage: We define the mutual coverage *m*_*c*_ between two domains $d_{1} \in \mathcal {D}_{1}$ and $d_{2} \in \mathcal {D}_{2}$ as their intersection length $L_{d_{1} \cap d_{2}}$ divided by the maximum length of the two domain lengths $L_{d_{1}}$ and $L_{d_{2}}$: $m_{c}(d_{1},d_{2}) = L_{d_{1} \cap d_{2}}/\max (L_{d_{1}},L_{d_{2}})$.
  The maximal value 1 of *m*_*c*_ is obtained when the two domains *d*_1_ and *d*_2_ are identical. Then, for each domain $d_{1} \in \mathcal {D}_{1}$, we define its best mutual coverage with $\mathcal {D}_{2}$ domains (${bm}_{c_{\mathcal {D}_{2}}}$) as its maximal mutual coverage with $\mathcal {D}_{2}$ domains: ${bm}_{c_{\mathcal {D}_{2}}} (d_{1}) = \max _{d_{2} \in \mathcal {D}_{2}}$
  (*m*_*c*_(*d*_1_,*d*_2_)). Sorting the $\mathcal {D}_{1}$ domains by size, we compute the mean best mutual coverage with $\mathcal {D}_{2}$ of groups of 50 $\mathcal {D}_{1}$ domains that we plot as a function of the mean length of the domains in the group. This results in an average mean best mutual coverage curve between domains in $\mathcal {D}_{1}$ and $\mathcal {D}_{2}$ as a function of $\mathcal {D}_{1}$ domain size.
- We say that a domain *d* has a match in $\mathcal {D}_{2}$ if ${bm}_{c_{\mathcal {D}_{2}}}(d) \geq 0.8$. $P_{\mathcal {D}_{2}}(\mathcal {D})$ is then defined as the proportion of domains $d \in \mathcal {D}$ that have a match in $\mathcal {D}_{2}$. Sorting the $\mathcal {D}_{1}$ domains by size, we consider them in groups $\mathcal {D}$ of 50 domains and plot $P_{\mathcal {D}_{2}} (\mathcal {D})$ as a function of the mean length of the domains in $\mathcal {D}$. This results in a matching proportion curve of domains in $\mathcal {D}_{1}$ and $\mathcal {D}_{2}$ as a function of $\mathcal {D}_{1}$ domain size.
- We say that a border *b* has a match in $\mathcal {B}_{2}$ when there is a border in $\mathcal {B}_{2}$ less than 100 kb away from *b* *i.e* ± 1 pixel away. $P_{\mathcal {B}_{2}}(\mathcal {B})$ is then defined as the proportion of borders $b \in \mathcal {B}$ that have a match in $\mathcal {B}_{2}$. Sorting the $\mathcal {B}_{1}$ borders according to their *associated lengths* (see below), we consider them in groups $\mathcal {B}$ of 100 borders and plot $P_{\mathcal {B}_{2}} (\mathcal {B})$ as a function of the average associated length of the borders in $\mathcal {B}$. This results in a matching proportion curve of borders in $\mathcal {B}_{1}$ and $\mathcal {B}_{2}$ as a function of $\mathcal {B}_{1}$ border associated length.

**Fig. 2:**
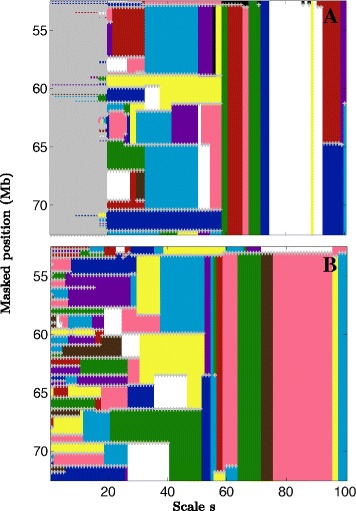
Multi-scale interval-communities. Multi-scale community structure along a 20 Mb long fragment of human chromosome 12 in IMR90 (**a**) and H1 ES (**b**) cell lines. Most (>99*%*) communities reduce to genomic intervals (see Section: Structural communities correspond to genome intervals), here we only represent these interval-communities. At each scale they are represented by colouring the segment from their masked start to masked end positions which are marked by *grey crosses* (+). Colours were limited to 10 for readability. When a community is found at 2 consecutive scales the same color is used

Domain length is an intuitive quantity to order a set of domains. In the same manner, we associated a length with each border of the genomic domains used in this work. TAD borders can be shared by at most 2 consecutive TADs, so we associated them with the length of the shortest TAD they border. At a fixed scale of analysis, a border of the novel interval-communities (see Section: Structural communities correspond to genome intervals) delimits two consecutive interval-communities, so (at that scale) we associated it with the minimum length of the two bordering communities. However these borders also present a strong pattern of conservation from one scale to another (Fig. 2; Additional file 1: Figure S5), so the largest of these lengths across the scales was retained as the final length associated with an interval-community border. In this way, border associated lengths allowed us to sort borders according to the importance (size) of the corresponding chromosome structures.

## Results and discussion

### Wavelet-based community detection in the DNA interaction network

**Fig. 3:**
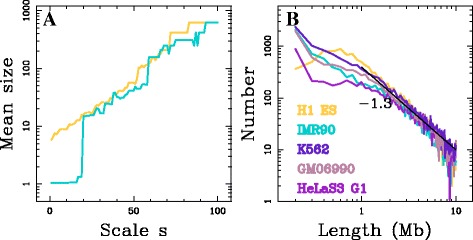
Multi-scale communities in the DNA interaction network. **a** Mean structural community size (in 100 kb pixels) for chromosome 12 as a function of the scale index in IMR90 (*blue*) and H1 ES (*yellow*). **b** Histogram of interval-communities genomic length (*l*) calculated in 100 kb bins in a log-log representation for different cell lines: IMR90 (*blue*), H1 ES (*yellow*), GM06990 (*pink*), K562 (*purple*) and HeLa (G1) (*light purple*). The *black straight line* correspond to the power-law behaviour *l* ^*α*^ with *α*=−1.3

As discussed above, Hi-C data can be represented as graphs where nodes represent DNA loci and the edges connect interacting loci, allowing us to reformulate the question of finding structural domains as a question of finding communities in the DNA interaction network. We used the fast implementation of the wavelet-based multi-scale community mining algorithm (Methods and Supplementary text: Graph wavelet transform and community mining in Additional file 1) with *η*=200 random Gaussian functions to estimate the distance correlation matrix. For each Hi-C dataset, we considered the 22 autosomes’ intra-chromosomal interaction networks constructed for non-overlapping 100 kb loci (Methods). We systematically applied the wavelet-based multi-scale community detection method to all the connected interaction networks scanning 100 scales logarithmically distributed in the range of available scales (Additional file 1: Equation (S13)) [40]. The average total running time per cell line was 5 h 40 mn using Matlab on a linux computing desktop with 8 Xeon CPU at 3.30 GHz. We first discuss the results obtained for human chromosome 12 in H1 ES and IMR90 cell lines as representative examples of the results obtained for all intra-chromosomal interaction networks. Chromosome 12 network initially contains 1 324 nodes. After the filtering procedure, 1 250 nodes are left in IMR90 and 1 249 in H1 ES (Methods). When applying the wavelet-based community detection method separately on the two interaction networks, we obtained 100 partitions of the masked genome for each cell line, one at each scale. Overall, we obtained 23 927 (resp. 4 266) communities for IMR90 (resp. H1 ES). As expected, the size of the resulting communities increases with the scale parameter (Fig. 3 a). For H1 ES the increase of the mean community size with the scale is homogeneous suggesting that there is no characteristic size for the community structure. For IMR90 we observe a first range of scales where the communities reduce to singletons (mean size ∼1), followed by an abrupt transition to a community mean size ∼17 (Fig. 3 a). The existence of singletons over a relatively large range of scales explains why the total number of communities in IMR90 is larger than in H1 ES. After removing the trivial communities (singletons), 3 342 (resp. 4 266) communities were kept in IMR90 (resp. H1 ES).

### Structural communities correspond to genome intervals

The interaction frequencies outside the diagonal blocks characterising the structural compartimentalisation as described in [11] are not negligible (look for instance at the region around [82,89] Mb in IMR90 that highly interacts with the region around [92,93] Mb in Fig. 1). This suggests that structural communities may not necessarily reduce to intervals along the genome. Hence for each non trivial community (community of size >1), we computed the proportion *P*_*int*_ of the largest set of successive 100 kb loci covered by the community over the size of the community: *P*_*int*_=1 when all the nodes of the community constitute an interval of the masked genome and *P*_*int*_=1/*N* where *N* is the size of the community when the community do not contain any pair of consecutive loci of the genome. Considering *P*_*int*_≥0.95 as a criterion for a community to constitute an interval along the genome, we observed for the 2 cell lines that more than 99% of the communities correspond to intervals of the genome. This property for the communities remains true for all the scales and whatever the size of the communities. This is consistent with the fact that at all scales, genomic neighbours tend to strongly co-localise resulting in higher frequency of interactions. These results demonstrate that the strongest motifs of structural organisation involve contiguous genomic segments. We will refer to the communities forming a genomic interval as *interval-communities*. We only kept the communities that correspond to an interval (*P*_*int*_≥0.95) reducing them to their main interval. This allowed us to adopt a simple representation of the structural-communities obtained across scales (Fig. 2). The differences observed between the resulting community size distributions in IMR90 and H1 ES (Fig. 3 a) are visible in this representation. We clearly see a first range of scales (*s*≤20) where the interval-communities reduce to singletons in IMR90 (Fig. 2 a) and not in H1 ES (Fig. 2 b). Above this critical scale, non trivial interval-communities appear in IMR90. Note that the mean size of the interval-communities for this first meaningful partitioning in IMR90 is larger than the ones observed in H1 ES for its first meaningful partitioning (smallest scale). This results in a lack of small non trivial interval-communities in IMR90.

### A hierarchical organisation of the genome

The representation in Fig. 2 reveals the hierarchical organisation of the communities. Across scales, small communities merge together to form bigger communities at larger scales. Hence the community borders present at the smallest scale progressively disappear at some larger scale allowing the emergence of bigger communities. Importantly, the conservation of borders from large scales to small scales is very high. For each pair of scales *s*2>*s*1, we computed the proportion of borders at the larger scale *s*2 that are also present at the smaller scale *s*1. This proportion is close to 1 regardless of the scales (Additional file 1: Figure S5). The fact that the borders are conserved across scales means that there is no “new” structure that emerges and that only existent ones merge together, i.e. small structures are nested into bigger ones. This is consistent with the results of recent studies suggesting that TADs hierarchically co-associate to form larger structures [16, 29, 46].

Another important property illustrated in Fig. 2 is the redundancy of the communities obtained across scales, underlining the robustness of the graph wavelet community mining protocol with respect to its stochasticity (usage of random vectors to estimate the graph wavelet correlation matrix; Methods). Hence, we kept only once each non trivial interval-communities (size ≥ 2 nodes and *P*_*int*_≥ 0.95). We also filtered out the communities that more than double in size when reintegrating the masked regions of the genome, e.g. interval-communities spanning the centromers. This leads to 386 (resp. 537) non trivial interval-communities in IMR90 (resp. H1 ES) for the chromosome 12. When applied to the 6 Hi-C datasets considered (Methods), the methodology presented for human chromosome 12 in H1 ES and IMR90 resulted in few thousands interval-communities per dataset (Table 1), except for the mitosis HeLaS3 dataset (discussed below). Interestingly, the length distributions of the interval-communities for the IMR90, GM06990, K562 and HeLaS3 G1 datasets are very similar, but they display differences with the one obtained for H1 ES dataset for small interval-communities (Fig. 3 b): there are more interval-communities involving only 2-3 nodes (200–300 kb) in the 4 differentiated cell lines datasets and a deficit in interval-communities of length ∼ 500 kb to ∼ 1.5 Mb relative to H1 ES. A possible interpretation of this excess of interval-communities of size ∼1 Mb in H1 ES, compared to differentiated cell lines, is that cell differentiation is accompanied by the merging of the small structural communities in a *structural consolidation* scenario. For larger communities, the interval-community size distributions in these 5 Hi-C datasets are almost identical. Indeed, for $l\gtrsim 2$ Mb, they display a power-law behaviour *l* ^*α*^ with *α*≃−1.3 (Fig. 3 b). Note that if communities of length ∼*l* would form a partition of the genome of length *L*, then the number of communities of this scale would be equal to *L*/*l* leading to *α*=−1 ($\gtrsim -1.3$). This underlines the existence of domains at all scales up to the chromosome length without a characteristic size for genome structuring.

**Table 1:** Number of structural communities

Cell line	N	N (filtered)	Remaining	Distinct
			communities	borders
H1 ES	12 343	65	12 278	5 751
IMR90	8 852	25	8 827	6 824
GM06990	10 279	60	10 219	6 967
K562	13 383	30	13 353	8 273
HeLaS3 G1	6 752	36	6 716	4 108
HeLaS3 M	1 059	4	1 055	885

For each cell line, N is the number of distinct non redundant and non trivial (size ≥ 2 i.e. 2 nodes) interval-communities. N(filtered) is the number of communities filtered out because *(i)* they do not correspond to a genomic interval or *(ii)* they double in size when going back to the original (not masked) positions. The last two columns correspond to the number of communities and distinct borders in the database

### Are interval-communities structural domains?

To test the robustness of the wavelet-based community detection method with respect to the possible absence of a community structure over some range of scales, we compared the interval-communities obtained for the Hi-C datasets in synchronised HeLaS3 cells during G1 and M phase, respectively (Methods). The original study [19] showed that the highly compartmentalised organisation described before from non synchronous cells [10, 11, 13, 15, 16, 20, 26, 27] was restricted to interphase and that during a cell cycle, chromosomes transit from a decondensed and spatially organised state during interphase to a highly condensed and morphologically reproducible metaphase chromosome state. In the former phase, the Hi-C interaction maps display similar plaid patterns of regional enrichment or depletion of long range interactions (as the one shown in Fig. 1) while the maps in mitotic cells change and the plaid patterns disappear [19]. For HeLaS3 G1 (resp. mitosis) dataset, we obtained 6 716 (resp. 1055) non trivial communities and 4 108 (resp. 885) distinct borders (Table 1). For the mitosis HeLaS3 Hi-C dataset, we obtained 1 059 communities from which we filtered out 4 resulting in 885 distincts borders (Table 1). Consistently with non synchronous cells, G1 cells present a hierarchical structure into interval-communities that increase in size across scales (Additional file 1: Figures S6 and S7). Small scale singletons hierarchically group to form large interval-communities at larger scales. As discussed above, the length distribution of the G1 HeLaS3 interval-communities is similar to the interval-communities size distribution obtained in the 3 other differentiated cell line datasets (Fig. 3 b). In contrast, metaphase chromosomes do not present a hierarchical structural organisation. More specifically, chromosomes 16, 21 and 22 do not present any structure (each node constitutes a community on the full available range of scales, Additional file 1: Figure S6). In the 19 other autosomes, at small scales each node is a singleton and above a critical scale a sharp discontinuity of the community sizes distribution is observed: nodes are abruptly grouped in a small number (2–5) of communities (Additional file 1: Figure S7). For 12 out of these 19 chromosomes, when divided in two communities, these communities correspond to the two chromosomal arms, as illustrated for chromosome 17 in Additional file 1: Figure S7. These results demonstrate that the wavelet-based community detection method does not produce misleading intermediate scale communities when no structuration exists in that scale range.

To strengthen this point in a noisy situation, we simulated a structural interaction matrix between 2000 nodes (comparable to the largest human chomosomes at resolution 100 kb) organised in fully connected interval-communities with no specific organisation at scales larger than the community size: the matrix is built as a series of 40 pairs of domains of size 20 nodes and 30 nodes with internal domain interaction set to 60, with the two first (resp. second) sub-diagonals set to 80 (resp. 70) to assure connectivity and with an additive Poisson noise over all interaction pairs of mean value *λ*=50 (Additional file 1: Figure S8 Left). When applying the graph wavelet community mining protocol, we recovered only trivial singleton communities at small and large scales. However in the intermediate scale range, we nicely recovered all the 20 and 30 nodes on a range of scales that depends on their size (Additional file 1: Figure S8 Right). This example shows that the method does not produce a fake hierarchical domain organisation by merging existing domains even in a noisy situation.

**Fig. 4:**
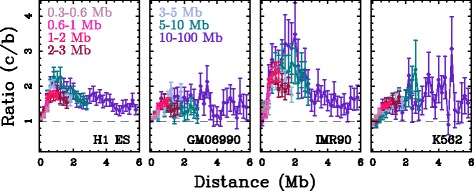
Are interval-communities structural domains? Ratio (*c*/*b*) of the number of interactions between two 100 kb loci that are inside the same community at equal distance from its center (*c*) and the number of interactions between loci in different communities at equal distance from a community border (*b*), versus the distance between them. Different colours correspond to different community size categories: 0.3 ≤*L*< 0.6 Mb (*light pink*), 0.6 ≤*L*< 1 Mb (*pink*), 1 ≤*L*< 2 Mb (*magenta*), 2 ≤*L*< 3 Mb (*dark pink*), 3 ≤*L*< 5 Mb (*light blue*), 5 ≤*L*< 10 Mb (*blue*) and 10 ≤*L*< 100 Mb (*purple*)

In order to verify that there are more interactions within interval-communities than between successive interval-communities, we compared the number of contacts between two 100 kb loci that are inside the same interval-community at equal distance from its center and the number of interactions between two loci at equal distance from one of the interval-community borders, as a function of the distance separating the pairs of loci. The ratio *vs* distance curves for different interval-community length categories show that on average there are more interactions within the communities than between communities, regardless of the cell line and the community length: the interaction ratio systematically increases to some maximal value at distances ∼ 1–2 Mb, from a maximal value ∼ 1.6 in GM06990 and K562, to ∼ 2.2 in H1 ES and ∼ 3 in IMR90. Over larger distances, the ratio remains rather constant in GM06990 and K562 and decreases to ∼ 1.5 in H1 ES and IMR90 (Fig. 4). This property holds true even for communities larger than 10 Mb. As a comparison, we performed the same analysis for the original TAD datasets in H1 ES and IMR90 (Methods). Over the shared domain length range, the interaction ratio *vs* distance curves computed for the TAD datasets present very similar shapes as observed for interval-communities (Additional file 1: Figure S9), reaching maximal values ∼3 in both H1 ES and IMR90. These results provide evidence that multi-scale interval-communities, very much like TADs, constitute units of 3D genome organisation bordered by structural barriers.

### Are TADs interval-communities?

**Fig. 5:**
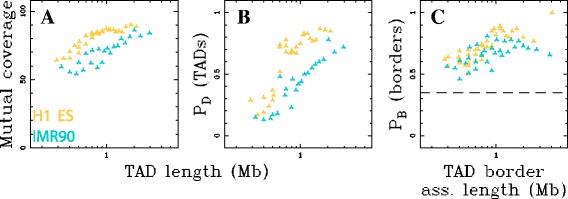
TADs are interval-communities. **a** Mean best mutual coverage of TADs with interval-communities, and (**b**) proportion of TADs that have a match in the interval-community database, as functions of the average TAD size (Methods). **c** proportion of TAD boundaries that have a matching interval-community border as a function of the average TAD border associated length (minimum of the length of the two bordering domains, Methods); only the set of interval-community borders with largest associated length covering 35% of the genome were used (see text); the *horizontal dashed line* marks this expected border matching proportion of 35%. In (**a**, **b**, **c**), *yellow marks* the analysis in H1 ES and *blue* in IMR90

We next compared our communities to the TADs previously described in H1 ES and IMR90 [11], asking to which extent the TADs and TAD borders are recovered in our hierarchical database of interval-communities. The mean best mutual coverage *vs* TAD length curve (Methods) between TADs and interval-communities is slightly higher in H1 ES as compared to IMR90 for all TAD lengths (Fig. 5 a), ranging from 62% (resp. 52%) at small length (300–500 kb) to 91% (resp. ∼ 89%) at larger length (∼ 1–2 Mb) in H1 ES (resp. IMR90). This suggests a good recovery of the largest TADs by the interval-community classification. Given the 100 kb resolution used in this analysis, it is not surprising to observe lower mutual coverages at small lengths where 1 pixel error results in a dramatic lowering of mutual coverage. We also observed that the proportion of TADs that have a matching structural community (Methods) increases with the domain length (Fig. 5 b). Only about 1/5 of the smallest TADs (≲ 500 kb) are recovered consistently with the fact that in this scale range a match has to be exact. For TADs longer than 1 Mb, the proportion of match is relatively high: in IMR90 it increases from 40% for TADs ∼1 Mb up to 70% for TADs ≥ 2 Mb and in H1 ES from 70% for TADs ∼ 1 Mb up to 85% for TADs of ∼ 2 Mb (Fig. 5 b). Comparison of TAD borders to interval-community borders shows good concordance for the two datasets (Fig. 5 c). For meaningful comparisons, we restricted the reference domain border set for each species to a subset of borders that at 100 kb resolution (±1 pixel) collectively cover no more than 35% of the genome. Interval-community borders with the largest associated lengths (Methods) are selected first. Given the overlap between borders at that resolution, this process resulted in selecting a different number of distinct interval-community borders in each species: 3 468 in H1 ES, 2 834 in IMR90, 3 171 in GM06990 and 3 478 in K562. TAD borders are recovered from 50% up to ∼ 90% in H1 ES and up to ∼80% in IMR90, depending on the TAD border associated length, while the expected recovery rate by chance is 35% (Fig. 5 c). These results quantify the high level of TAD recovery by interval-communities for domain length $\gtrsim $ 1 Mb. Altogether, these results show that there is a significant agreement between TADs and the interval-communities. This provides evidence that interval-communities captures similar organisation principle of genome structure and, thus, extends this description up to chromosome size.

To test the robustness of our methodology with regards to the binning resolution, we reproduced the analysis of the IMR90 data at a finer (40 kb, total running time 8h48mn) and a coarser (200 kb, total running time 31 mn) resolution (Additional file 1: Figure S10). The lengths of interval-communities determined at these 2 resolutions nicely reproduce the distribution obtained at the 100 kb resolution (Additional file 1: Figure S10A). Intervals-communities of size $\gtrsim 1-2$ Mb strongly match between these intervals communities datasets (recovery proportion ≃70−90*%*, Additional file 1: Figure S10B). For smaller community size, recovery proportion between datasets decreases with community size in the same manner for the 3 resolution pairs. This can be understood when noting that the isolation strength of community borders significantly weakens when decreasing the genomic distance below ∼1 Mb (Fig. 4). Finally, the proportion of TADs that have a match in the interval-community database is similar at 40 kb resolution than at 100 kb resolution (Additional file 1: Figure S11). This demonstrates that the results do not depend on the choice of the 100 kb resolution and further underlines that the lower structural domain recovery rate generally observed for small domain sizes (≲1 Mb) is likely related to the weaker isolation strength of structural domain borders over short distances (≲1 Mb).

### Conservation of structural communities across cell lines

**Fig. 6:**
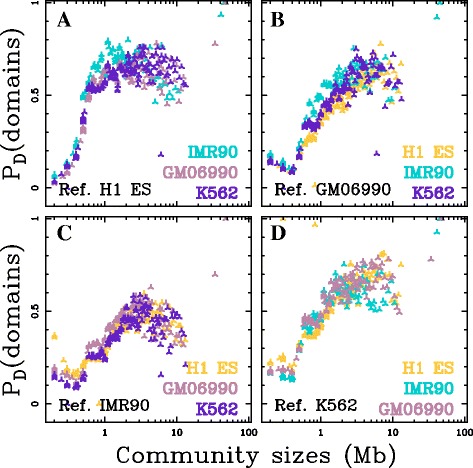
Conservation of interval-communities between cell lines. Proportion of interval-communities in the query cell lines H1 ES (*yellow*), IMR90 (*blue*), GM06990 (*pink*) and K562 (*purple*) that have a matching interval-communities in the reference cell line indicated in each plot: H1 ES (**a**), GM06990 (**b**), IMR90 (**c**) and K562 (**d**). Proportion of interval-community matches is computed over groups of 50 query interval-communities ordered by length (Methods)

In the pioneering study [11], TADs were described to be conserved between cell lines. We observed that interval-communities in different cell lines present similar size distributions (Fig. 3 b). This led us to investigate to which extent they are conserved across cell lines. To compare the communities obtained in different cell lines, we used each of the interval-community database obtained in H1 ES, GM06990, IMR90, K562, as a reference domain set and computed the proportion of matching interval-communities of the 3 other cell lines relative to this reference set (Methods). We observed that small interval-communities (≲ 600 kb) are not well conserved between different cell lines (Fig. 6). This might result from the fact that Hi-C data are average over cell populations and that some regions may present different structural organisations from cell to cell blurring the insulator property of structural domain borders at small scales. However, when considering interval-communities of larger sizes, higher conservation was observed (Fig. 6). More than 60% of intervals-communities of length $L \gtrsim $ 0.6 Mb in the differentiated cell lines correspond to an interval-community in H1 ES (Fig. 6 a). H1 ES interval-community dataset thus contains a large proportion of the interval-communities observed in the differentiated cell lines above ∼ 600 kb. When using one differentiated cell line interval-community database as reference, we observed a maximal recovery rate that is similar for the 3 other cell lines: 45% for sizes $\gtrsim $ 2 Mb in IMR90, 65% for sizes $\gtrsim $ 1.5 Mb in GM06990 and 70% for sizes $\gtrsim $ 1.5 Mb in K562 (Fig. 6 b, c and d). The observed differences likely reflect the excess of interval-communities in the size range 0.5-1.5 Mb observed in H1 ES relative to the differentiated cell lines (Fig. 3 b). As a comparison, we performed the same analysis for the TADs that were claimed to be conserved between H1 ES and IMR90 cell lines [11] (Additional file 1: Figure S12). Like for interval-communities, the correspondance between TADs in the two cell lines decreases for domain sizes ≲ 600 kb. For larger domain sizes, we observed that H1 ES TAD dataset contains more (maximal value ∼60%) of the IMR90 TADs than the IMR90 TAD dataset contains H1 ES TADs (∼45%). These results corroborate the conservation of structural domains of length ∼1–2 Mb between cell lines in the 45–70% range but also extend this conservation to the largest interval-communities up to length $\gtrsim $ 10 Mb.

## Conclusions

We introduced a fast multi-scale community mining algorithm based on spectral graph wavelets [40] to identify structural motifs from high-throughput chromatin conformation capture data (Hi-C) [10]. Hi-C data were represented as intra-chromosomal interaction networks and structural motifs were delineated as communities of these networks. The novelty of this approach relies on the combination of a multi-scale procedure and a representation of the data that is independent of the exact assembly of the reference genome over length scales larger than the window size used to construct the interaction network. The proposed methodology has no a priori on the size and on the nature of the structural motifs. The application of this protocol to 6 Hi-C datasets led to a database of several thousands structural communities (Table 1). The database of interval-communities in mitotic HeLaS3 cells that were described not to present a TAD-like structural organisation [19], does not contain any intermediary scale structural communities, illustrating the robustness of the proposed methodology with regards to the absence of structural motifs. Consistently with the recent usage of Hi-C data for genome sequence assembly [47, 48], we observed that structural-communities in unsynchronised and G1 cells form hierarchies of chromosome intervals of length ranging from the resolution (100 kb) to the chromosome lengths ($\gtrsim 10$ Mb) (Fig. 3). The prevalence of interval-communities underlines that chromosome folding is mainly driven by interactions between neighbouring loci, at all scales of observation. This constitutes a justification that TAD-like structural motifs indeed correspond to chromosome intervals. For domains significantly larger than the resolution of the analysis ($\gtrsim 600$ kb), a majority of the TADs [11] are recovered as interval-communities (Fig. 5) and, whatever the interval-community length, their borders present an insulator-like behaviour (Fig. 4) as expected for TAD-like structural motifs. Hence interval-communities capture similar structural organisation patterns as TADs but over the *full* chromosome range of scales.

This novel multi-scale structural decomposition of human chromosomes provides an original framework to question structural organisation and its relationship to functional regulation. It allowed us to reformulate the question of structural domain conservation between different cell lines across the scales: a high level of structural conservation between cell lines up to the largest scales becomes apparent. For example, ∼ 65% of the differentiated cell lines interval-communities larger than 600 kb were also found to be structural-communities in H1 ES cell line (Fig. 6 a). It was previously noted that there likely exists some links between structural domains and replication domains [23, 25, 27, 49] including the so-called replication timing U-domains [24, 50]. U-domains are bordered by early replicating *master* replication initiation zones that present similar insulating properties as the ones observed for TADs and interval-communities borders (Fig. 4 and Additional file 1: Figure S9) [24]. In Human ES cells, master replication initiation zones are enriched in CTCF and pluripotent transcription factors NANOG and OCT4 that were recently shown to contribute to the overall folding of embryonic stem cells genome via specific long-range contacts [51, 52], and appear to be fundamental determinants of pluripotency maintenance [53, 54]. In particular they are at the heart of the so-called consolidation phenomenon [17, 23, 55, 56] corresponding to early to late transitions from embryonic stem cells to differentiated cells coinciding with the emergence of compact heterochromatin at the nuclear periphery [54]. ES cell line are characterised by smaller replication U-domains [24]. Here we observed in H1 ES cell line an excess of interval-communities in the range of scales from ∼500 kb to ∼1.5 Mb as compared to the differentiated cell lines (Fig. 3 b). These domains not observed in differentiated cell lines might be subject to some structural consolidation scenario during cell differentiation, similar to the one described for replication timing domains. For example, the strutural community border present in H1 ES and absent in IMR90 at position ∼84 Mb in Fig. 1 correspond to a replication timing U-domain border specific of ES cell line. Further analysis of the structural consolidation scenario is likely to shed a new light on the role of structural organisation in the epigenetically regulated chromatin reorganisation that underlies the loss of pluripotency and lineage commitment [54]. It was shown that master origins of replication conserved between 6 cell lines are encoded in the DNA sequence via a local enrichment in nucleosome excluding energy barriers [57, 58]. This raises the question whether borders of the conserved structural community borders (Fig. 6) might be specified by a similar genetic mechanism.

A recent Hi-C experimental study at much higher (kb) resolution has provided some refined partitioning of the human genome by TADs of mean size ∼180 kb [20], much closer to the estimate ∼100-kb previously reported in *Drosophila* [16]. Interestingly, as in *Drosophila*, these refined TADs seem to have some specific epigenetic chromatin identity that can change dramatically their functional identity in different cell types [16, 20, 59]. Detecting interval-communities at higher resolution can provide better quantification of the chromatin state blocks as epigenetic communities. The wavelet-based community detection method provides us with a tool to investigate further the existence of some underlying rules for the association of structural/functional domains across scales. The robustness of the proposed protocol with respect to rearranged genomes is a key property to pursue this research.

## Additional file

Additional file 1 Supplementary Online Material. Additional data file 1 contains supplementary text: Graph wavelet transform and community mining, supplementary Figures S1 to S12 and supplementary Table S1 (PDF). (PDF 3963 kb)

## Abbreviations

DNA: Deoxyribonucleic acid
Hi-C: High-throughput 3C
H1 ES: Embryonic stem cell line H1
TAD: Topologically associating domains
3C: Chromatin conformation capture
