# Supplementary material for: Multi-scale structural community organisation of the human genome
Source: BMC Bioinformatics. 2017 Apr 11;18:209. doi: 10.1186/s12859-017-1616-x (PMC5387268; doi:10.1186/s12859-017-1616-x)
Supplement: Additional file 1 — Supplementary Online Material. Additional data file 1 contains supplementary text: Graph wavelet transform and community mining, supplementary Figures S1 to S12 and supplementary Table S1 (PDF). (PDF 3963 kb) [file 12859_2017_1616_MOESM1_ESM.pdf]

Supplementary data for
Multi-scale structural community organisation of the human genome

Rasha E. Boulos [1,†], Nicolas Tremblay [1,‡], Alain Arneodo [1,§], Pierre Borgnat [1] and Benjamin Audit [1,*]

[1]Univ Lyon, Ens de Lyon, Univ Claude Bernard Lyon 1, CNRS, Laboratoire de Physique, F-69342 Lyon, France
[†] present address: Montpellier Cancer Institute (ICM), Montpellier Cancer Research Institute (IRCM) Inserm U1194, University of Montpellier, Montpellier, France
[‡] present address: INRIA Rennes - Bretagne Atlantique, Beaulieu Campus, Rennes, France and Institute of Electrical Engineering, EPFL, Lausanne, Switzerland
[§] present address: LOMA, Université de Bordeaux, CNRS, UMR 5798, 51 Cours de le Libération, 33405 Talence, France

Contents

Supplementary text: Graph wavelet transform and community mining 2

Supplementary figures 8

Supplementary table 15

References 16

[*]To whom correspondence should be addressed. Tel: +33 4 7272 8691; Email: benjamin.audit@ens-lyon.fr

Supplementary text: Graph wavelet transform and community mining

We propose here a short introduction to signal processing on graphs, in particular the spectral graph wavelet transform, and its application to graph community mining. Graph communities are major graph structures described as groups of nodes highly connected between them and less connected with the rest of the graph. This supplementary text does not contain original material, it is intended to provide the reader with the background information to fully apprehend the proposed analysing approach of Hi-C data, as well as, references to the original bibliography.

Networks have become essential to represent data from a variety of complex systems in social sciences (Wasserman and Faust, 1994; Scott, 2000), biology (Rives and Galitski, 2003; Spirin and Mirny, 2003; Mendes and Dorogovtsev, 2003), computer sciences (Mendes and Dorogovtsev, 2003; Pastor-Satorras and Vespignani, 2004), engineering and many other areas of fundamental and applied sciences (Albert and Barabasi, 2002; Boccaletti *et al.*, 2006; Caldarelli and Vespignani, 2007). These networks can be represented as *graphs* (West, 1995; Bollobas, 1998; Bondy and Murty, 2008; Diestel, 2010), mathematical objects where the elements of study are represented as *nodes* (or *vertices*) and the connections between them constitute the edges of the graph. A graph $G = (V, E)$ is defined by a set of nodes V and a set of *edges* $E \subset V^2$ that link the nodes to each other. Here, we only consider finite graphs where edges are not directed (undirected graphs) and with no loop (node self-connection). We note $n = |V|$, the number of nodes and $m = |E|$, the number of edges. Matrices are powerful tools for representing graphs in a computer and developing mathematical tools to study the associated graph properties (West, 1995; Chung, 1997; Bollobas, 1998; Bondy and Murty, 2008; Diestel, 2010). The adjacency matrix A of a graph G is a $n \times n$ matrix where the entries $a_{ij} = 1$ if the nodes x_i and x_j are connected (adjacent), and $a_{ij} = 0$ otherwise. Note that the adjacency matrix of an undirected graph is symmetric. When assigning a weight to each edge the graph becomes *weighted* and the weighted adjacency matrix $W = (w_{ij})$ has non-null values when x_i and x_j are connected and 0 otherwise: the higher the value of w_{ij}, the stronger the link between them. We note M the adjacency (A) or the weighted adjacency (W) matrix.

In applications such as the ones cited above, one may be interested in analysing the distribution of data values residing on the vertices of the graph, such data sets have been described as *signals on graphs*. Naturally, one can wonder what are the best strategies to characterise and to extract efficiently the information from these signals on graphs. Recent developments in the area of graph-signal processing provide us with operators to analyse signals on graphs (Shuman *et al.*, 2013); they generalise classical signal expansions, such as Fourier and wavelet decompositions, to the graph signal setting. Importantly, the graph Fourier modes and the graph wavelets depend on the graph that is considered and, thus, convey information about the graph topology. This property can be used to build graph community mining methods (Fortunato, 2010; Tremblay and Borgnat, 2014). For example, *Spectral clustering*, in its simplest form, defines a bipartition of a graph based on the sign of the graph Fourier mode of lowest, non zero frequency (Fiedler, 1973; von Luxburg, 2007) and was previously applied to the Hi-C interaction network (Chen *et al.*, 2015). Here, we present the construction of spectral graph wavelets (Hammond *et al.*, 2011) and their usage to

build a fast multi-scale community mining algorithm (Tremblay and Borgnat, 2014).

Graph spectral domain

In the area of signal processing on graphs, spectral graph theory has become a tool to define frequency spectra and expansion bases to define graph Fourier transforms. We present the construction of graph Fourier basis as the eigenvectors of the Laplacian matrix of the graph (Shuman *et al.*, 2013) from which the spectral graph wavelets we will be using are constructed (Hammond *et al.*, 2011). Note that alternative constructions of Fourier modes have also been explored (Sandryhaila and Moura, 2012).

We first recall some spectral graph theory elements (Chung, 1997). Let $G = (V, E)$ be an undirected and connected graph with $M = A$ or W, the adjacency or weighted adjacency matrix corresponding to the graph. A signal (or a function) $F : V \longrightarrow \mathbb{R}$ on the nodes of the graph can be seen as a column vector $F \in \mathbb{R}^n$, where the ith component F_i of the vector F represents the signal value at node x_i. The graph *Laplacian matrix* L is defined as: $L = D - M$, where D is a diagonal matrix whose element $d_{ii} = \Sigma_j m_{ij}$, is the degree (or weighted degree *i.e.* strength) of the node. Note that the Laplacian matrix can also be found under its normalised form: $\mathcal{L} = D^{-\frac{1}{2}} L D^{-\frac{1}{2}}$. In the case of a weighted graph, the non-normalised graph Laplacian is also known as the combinatorial graph Laplacian. In all cases, the graph Laplacian is a real symmetric matrix (because M is symmetric), and hence, it has a complete set of orthonormal column eigenvectors, denoted $\{\chi_l\}_{l=0,..,n-1}$. These graph eigenfunctions have associated positive eigenvalues $\{\lambda_l\}_{l=0,..,n-1}$. Zero appears as an eigenvalue with multiplicity equal to the number of connected components. Since we are only interested in connected graphs, we consider the Laplacian eigenvalues ordered as $0 = \lambda_0 < \lambda_1 \leq \lambda_2 ... \leq \lambda_{n-1} := \lambda_{max}$. The set of λ_i's is called the *spectrum* of L (or spectrum of the associated graph G). We note $\chi = (\chi_0|\cdots|\chi_l|\cdots|\chi_{n-1}) = (\chi_{il})$.

Let us recall that the *classical Fourier transform*

$$\hat{f}(\xi) = \int_R f(t) e^{-2\pi i \xi t} dt, \tag{S1}$$

is the projection of a function f on the complex exponentials, which are the eigenfunctions of the one dimensional Laplace operator: $-\Delta(e^{2\pi i \xi t}) = -\frac{d^2}{dt^2} e^{2\pi i \xi t} = (2\pi\xi)^2 e^{2\pi i \xi t}$. When considering the circular graph of n nodes * that is nothing but the regular (discrete) line with periodic boundary conditions, L is the classical discrete Laplace operator *i.e.* the discrete second derivative operator, and its eigenvectors are the usual discrete Fourier modes. Following this fundamental analogy between the classical case and the graph setting, the eigenvectors ($\{\chi_l\}_{l=0,..,n-1}$) of the graph Laplacian of graph G are interpreted as the graph Fourier modes associated to frequencies $\sqrt{\lambda_l}$ for that graph. Consequently, the *Graph Fourier Transform* (GFT) $\hat{F}$ of a signal F on the vertices of a graph G is defined as the projection of F on its graph Fourier modes : $\hat{F}_l = \sum_{i=1}^{n} F_i \chi_{il}$, which can be written in matrix notation as:

$$\hat{F} = \chi^{\top} F, \tag{S2}$$

*Nodes are labelled from 0 to $n-1$ and node i is connected to the two nodes $i-1 \pmod{n}$ and $i+1 \pmod{n}$.

where $\chi^\top$ is the transpose of χ. In the same manner, the *classical inverse Fourier transform*:

$$f(t) = \int_R \hat{f}(\xi)e^{2\pi i\xi t}dt, \tag{S3}$$

is the reconstruction of f as a weighted sum of the Fourier vectors, which is mimicked in the graph setting as: $F_i = \sum_{l=0}^{n-1} \hat{F}_l \chi_{il}$, that can be written in matrix notation as:

$$F = \chi\hat{F}. \tag{S4}$$

In this construction, signal processing on graphs can be considered as a generalisation of the "classical" discrete signal processing, which is recovered when considering circular graphs. Figure S2 illustrates some Fourier modes on a hierarchical toy graph (Fig. S1). One can already remark that the first eigenvectors are very informative for community detection as discussed above. For instance, partitioning the toy graph according to the sign of χ_1, leads to 2 meaningful communities (Fig. S2A).

Spectral graph wavelets and the graph wavelet transform

There is not a unique way to extend the notion of wavelets to the graph setting. For example, graph wavelets have been defined using the notion of diffusion in the node neighbourhood (Coifman and Maggioni, 2006), lifting (Jansen *et al.*, 2009), and filter banks (Narang and Ortega, 2012). Here, we present the construction of graph wavelets using the graph spectral domain (Hammond *et al.*, 2011). These graph wavelets have been shown to be well adapted to the problem of community mining (Tremblay and Borgnat, 2014).

The classical wavelet transform (WT) is a space-scale analysis which consists in expanding signals in terms of wavelets which are constructed from a single function, the "analysing wavelet" ψ, by means of translations and dilations. The WT of a real-valued function f is defined as (Mallat, 1998):

$$T_\psi[f](x_0, s) = \frac{1}{\sqrt{s}} \int_{-\infty}^{+\infty} f(x)\psi(\frac{x_0 - x}{s})dx \ , \tag{S5}$$

where x_0 and s (> 0) are the space and scale parameters respectively. At a fixed scale s, Eq. (S5) can be interpreted as the convolution of the signal with the wavelet ψ_s centered on 0 and derived from the analysing wavelet ψ as: $\psi_s(t) = \frac{1}{\sqrt{s}}\psi(\frac{t}{s})$. Importantly, given the spectral perspective of our construction, convolution in the direct space of a signal f by a filter h corresponds to multiplication in the Fourier space: $\widehat{(h * f)}(\xi) = \hat{f}(\xi)\hat{h}(\xi)$. Mimicking this property, convolution of the graph signal F by the graph filter H can be defined as the inverse Fourier transform (Eq. (S4)) of the product of the graph Fourier transform $\hat{H}$ and $\hat{F}$ of F and H, respectively:

$$H * F = \chi(\hat{H} \circ \hat{F}), \tag{S6}$$

where $\circ$ stands for the component wise column multiplication. Mimicking the fact that the Fourier transform of ψ_s can be written as follows: $\hat{\psi}_s(\xi) = \sqrt{s}\hat{\psi}(s\xi)$, we introduce a continuous function $g : \mathbb{R}^+ \to \mathbb{R}^+$ equivalent to the band-pass filter $\hat{\psi}$, and we construct the graph Fourier transform

$\hat{H}^{(s)}$ of the graph wavelet filter at scale s as follows:

$$\hat{H}^{(s)}_{k+1} = \sqrt{s} g(s\lambda_k), \qquad \forall k \in [0, n-1]. \tag{S7}$$

This results in the following definition of the graph wavelet transform of a graph function F: $W^{(s)}[F] = \chi(\hat{H}^{(s)} \circ \hat{F})$. Denoting $G^{(s)}$ the filter matrix at scale s in the graph Fourier space defined by:

$$G^{(s)} = \mathrm{diag}\left(g(s\lambda_0), \dots, g(s\lambda_{n-1})\right) = \begin{bmatrix} g(s\lambda_0) & \cdots & 0 \\ \vdots & \ddots & \vdots \\ 0 & \cdots & g(s\lambda_{n-1}) \end{bmatrix}, \tag{S8}$$

and using Eq. (S2), the definition of the graph wavelet transform of F can be written as:

$$W^{(s)}[F] = \sqrt{s}\chi G^{(s)} \chi^{\top} F. \tag{S9}$$

Let $\Delta^{(k)}$ denote the Dirac function on the graph centred on the node x_k: $\Delta^{(k)}_i = 1$ on node x_k and $\Delta^{(k)}_i = 0$ otherwise. The graph wavelet centred on node k at scale s then reads: $\Phi^{(s,k)} = W^{(s)}[\Delta^{(k)}] = \sqrt{s}\chi G^{(s)} \chi^{\top} \Delta^{(k)}$. Since the matrix $(\Delta^{(1)}|...|\Delta^{(n)})$ is the identity matrix, we can write the matrix $\Phi^{(s)}$ of all the wavelets at scale s as the following simple matrix product:

$$\Phi^{(s)} = (\Phi^{(s,1)}| \cdots |\Phi^{(s,n)}) = \sqrt{s}\chi G^{(s)} \chi^{\top}. \tag{S10}$$

For a given graph, the graph wavelets $\Phi^{(s)}$ are thus entirely determined by the band-pass kernel filter g. We use the filter $g(x)$ proposed in Tremblay and Borgnat (2014) that is band-pass in the range $[1, \frac{1}{\lambda_1}]$ and that is well conditioned for the task of community detection over the scale range $[s_{min} = \frac{1}{\lambda_1}, s_{max} = \frac{1}{\lambda_1^2}]$. The above construction of graph wavelets does not guarantee their proper normalisation, so that the $\sqrt{s}$ normalisation factor can be dropped. Note, however, that normalisation is not required for the multi-scale community mining protocol described below. When required this can be remedied by a posteriori normalisation of the graph wavelets.

The graph wavelet at a reference node x_k gives an idea on how the graph is perceived from that node. As illustrated in Figure S3 for our hierarchical toy graph, at small scales, the graph wavelet coefficients are higher in the “close” neighbourhood of the reference node and for larger scales the graph wavelet extends over a larger neighbourhood. At small scale, the graph wavelet has an “ego-centered” view of the graph, it takes the value 1 at the reference node and 0 elsewhere (like a Dirac) (Fig. S3A). At larger scales, the graph wavelet coefficients expend on the neighbourhood of the reference node (Fig. S3B-D), reflecting the graph topology. In Figure S3B, the graph wavelet coefficients are positive over the full 16 node group around the reference node, reflecting the second level of organisation of the graph in 8 groups of 16 nodes (Fig. S1B). In the same manner, at a larger scale, the positive values of the graph wavelet coefficients (Fig. S3C,D) recover the 32 (resp. 64) node group of the reference node at the third (resp. fourth) level of the hierarchical organisation of the toy graph (Fig. S1C,D) .

Multi-scale community mining using graph wavelets

The intuition behind the community mining method based on graph wavelet proposed by Tremblay and Borgnat (2014), is to consider that the wavelet centred around a node is an "ego-centered" vision of the graph, and that two nodes in the same community are expected to have a very similar view of the graph (Fig. S3). In other words, and this is the central idea of the algorithm, nodes are classified together if their neighbourhood as defined by graph wavelets are similar. The method consists at each scale s in three steps (Tremblay and Borgnat, 2014):

- For each node x_k, one defines its feature vector as the coefficients of the wavelet $\Phi^{(s,k)}$ that encodes local information on the graph topology seen by the node x_k (Fig. S3). Spectral graph wavelets $\Phi^{(s,k)}$ are derived from the normalised Laplacian $\mathcal{L}$.

- To define to which extent two nodes x_k and $x_{k'}$ have a similar environment, a distance matrix $\mathcal{D}^{(s)}$ is created where the correlation distance $\mathcal{D}^{(s)}(k,k')$ between nodes x_k and $x_{k'}$ is one minus the correlation $\mathcal{C}^{(s)}(k,k')$ between the wavelets $\Phi^{(s,k)}$ and $\Phi^{(s,k')}$ (Fig. S4):

$$\mathcal{C}^{(s)}(k,k') = \frac{{\Phi^{(s,k)}}^\top \Phi^{(s,k')}}{||\Phi^{(s,k)}||_2||\Phi^{(s,k')}||_2} \quad \text{and} \tag{S11}$$

$$\mathcal{D}^{(s)}(k,k') = 1 - \mathcal{C}^{(s)}(k,k'), \qquad \forall (k,k') \in [1\dots n]^2. \tag{S12}$$

  Note that this distance measure is independent of graph wavelet normalisation.

- A hierarchical clustering algorithm is used to classify the nodes (Fig. S4). The hierarchical algorithm outputs a dendrogram that needs to be "cut" to obtain a partition $P^{(s)}$. To cut the dendrogram, the method defines a criterion based on averaging the maximal gaps of all the root-leaf paths of the dendrogram. For each node x_k, one computes the gap function Γ_k as the path length between the leaf corresponding to node x_k and the begining of the dendrogram. Then after averaging all gaps functions into a global gap function, the best cut corresponds to the maximum of this global gap function (Tremblay and Borgnat, 2014).

Repeating these three steps for a set of scales $(s_i)_{i\in[1\dots N_{scale}]}$ (i is called the *scale index*), one obtains a multi-scale set of partitions $P^{(s_i)}$ of the nodes (Tremblay and Borgnat, 2014). Scales s_i are typically chosen to be logarithmically distributed in the range $[s_{min}, s_{max}]$:

$$s_i = s_{min}\left(\frac{s_{max}}{s_{min}}\right)^{(i-1)/(N_{scale}-1)}. \tag{S13}$$

Fast multi-scale graph wavelet community mining

The major inconvenient of the graph wavelet community mining protocol is the computation cost. Two approximations have been proposed allowing this protocol to be suitable for the analysis of large graphs ($\gtrsim 10000$ nodes) (Tremblay and Borgnat, 2014).

On the one hand, instead of computing the graph wavelets centred on the n nodes of the graph which requires n wavelet transforms of Dirac functions (Eq. (S10)), the matrix of correlations $\mathcal{C}^{(s)}(k,k')$ between graph wavelets at scale s (Eq. (S11)) is estimated using the graph wavelet transforms $W^{(s)}[R^{(j)}]$ of η ($\ll n$) random Gaussian functions on the graph $(R^{(j)})_{j\in[1\dots\eta]}$

(Tremblay and Borgnat, 2014). More specifically, given a random Gaussian vector R, it can be shown that the correlations between its graph wavelet coefficients (the projections of R on the graph wavelets: $W^{(s)}[R]_k = \Phi^{(s,k)^\top} R$) reduce to the correlation between graph wavelets: $\mathrm{corr}(W^{(s)}[R]_k, W^{(s)}[R]_{k'}) = \mathcal{C}^{(s)}(k,k')$. Hence, given η realisations $(R^{(j)})_{j\in[1\ldots\eta]}$ of R, $\mathcal{C}^{(s)}(k,k')$ can be estimated by the sample correlation coefficient between the vectors $(W^{(s)}[R^{(j)}]_k)_{j\in[1\ldots\eta]}$ and $(W^{(s)}[R^{(j)}]_{k'})_{j\in[1\ldots\eta]}$ (Tremblay and Borgnat, 2014).

On the other hand, the graph wavelet transform can be computed using the fast algorithm proposed in Hammond *et al.* (2011). Eq. (S9) shows that the calculation of the graph wavelet transform at a scale s requires the knowledge of the graph Fourier matrix χ, itself calculated by the diagonalisation of the graph Laplacian. The diagonalisation of a matrix of size n typically needs a calculation time cubic in the number of nodes n, which makes it impracticable to use for graphs with more than a few thousand nodes. To overcome this difficulty and to calculate the wavelet transform of a signal F quickly, it is in fact possible, using an approximated algorithm, to avoid the explicit calculation of the graph Fourier matrix (Hammond *et al.*, 2011). This approach consists in approximating each filter $g(s.)$ into a truncated Chebyshev polynomial of degree p:

$$g(sx) \simeq \sum_{i=1}^{p} \alpha_i^{(s)} x^i, \qquad \forall x \in \mathbb{R}^+. \tag{S14}$$

From Eqs. (S8) and (S14), it results the following approximation of the matrix $G^{(s)}$

$$G^{(s)} \simeq \sum_{i=1}^{p} \alpha_i^{(s)} \Lambda^i, \tag{S15}$$

where $\Lambda = \mathrm{diag}(\lambda_0, \ldots, \lambda_{n-1})$ is the diagonal matrix whose diagonal entries are the eigenvalues λ_k of the Laplacian matrix L. Observing that $\chi\Lambda^i\chi^\top = L^i$, we can write the following approximations for the construction of graph wavelets and the computation of the graph wavelet transform:

$$\Phi_s \simeq \sum_{i=1}^{p} \alpha_i^{(s)} L^i, \tag{S16}$$

and

$$W^{(s)}[F] \simeq \sum_{i=1}^{p} \alpha_i^{(s)} L^i F. \tag{S17}$$

Hence, instead of having to calculate the diagonalisation of L, one can compute the graph wavelet transform $W^{(s)}[F]$ only via matrix-vector multiplications, given that L^i, $i = 1, ..., n$ have to be computed only once per graph. The fast graph wavelet community mining protocol relies on this approximation to compute the graph wavelet transforms of the random Gaussian functions required to compute the distance matrix $\mathcal{D}^{(s)}$.

Supplementary figures

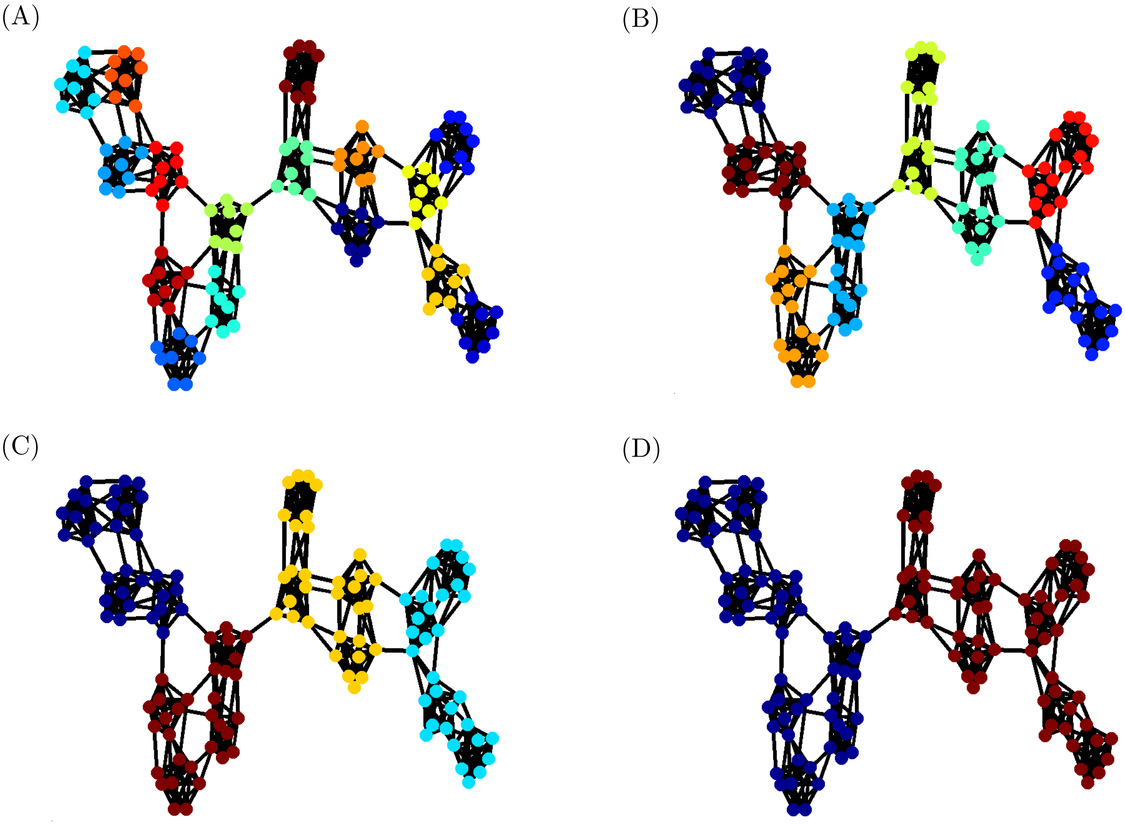

Figure S1. Multi-scale community structure. A toy graph of 128 nodes built with a hierarchical structure at 4 scales. (A) At the smallest scale, 16 groups of 8 nodes are fully linked together. (B) Then pairs of groups are linked together by 4 edges, resulting in a structure of 8 groups of 16 nodes. (C) Pairs of 16 nodes groups are connected with 4 edges, resulting in a structure of 4 groups of 32 nodes. (D) Pairs of 32 nodes groups are connected by 2 edges, resulting in 2 groups of 64 nodes, that are connected together with only one edge, in order to obtain a connected graph. This way of construction ensures that, at each scale, there is more edges inside each group than in between the groups. This general form of hierarchical graphs (Sales-Pardo *et al.*, 2007) is widely used as a benchmark for multi-scale community mining tools. The graph can be easily partitioned into 16 (A), 8 (B), 4 (C) and 2 (D) communities: from small (A) to large (D) scales. At each scale, each colour corresponds to a community.

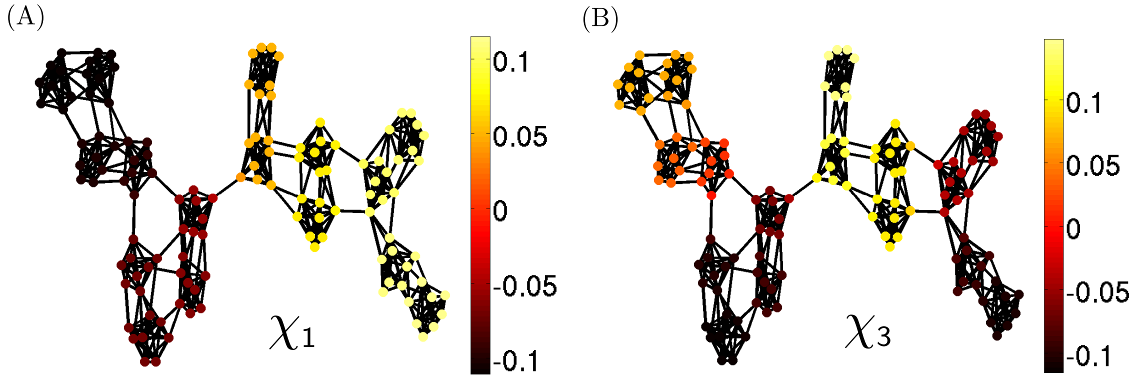

Figure S2. Graph Fourier modes. Graph Fourier modes χ_1 (A) and χ_3 (B) of the toy graph with 128 nodes (Fig. S1). Nodes are colour coded according to the Fourier mode values on each node using the colour maps at the right.

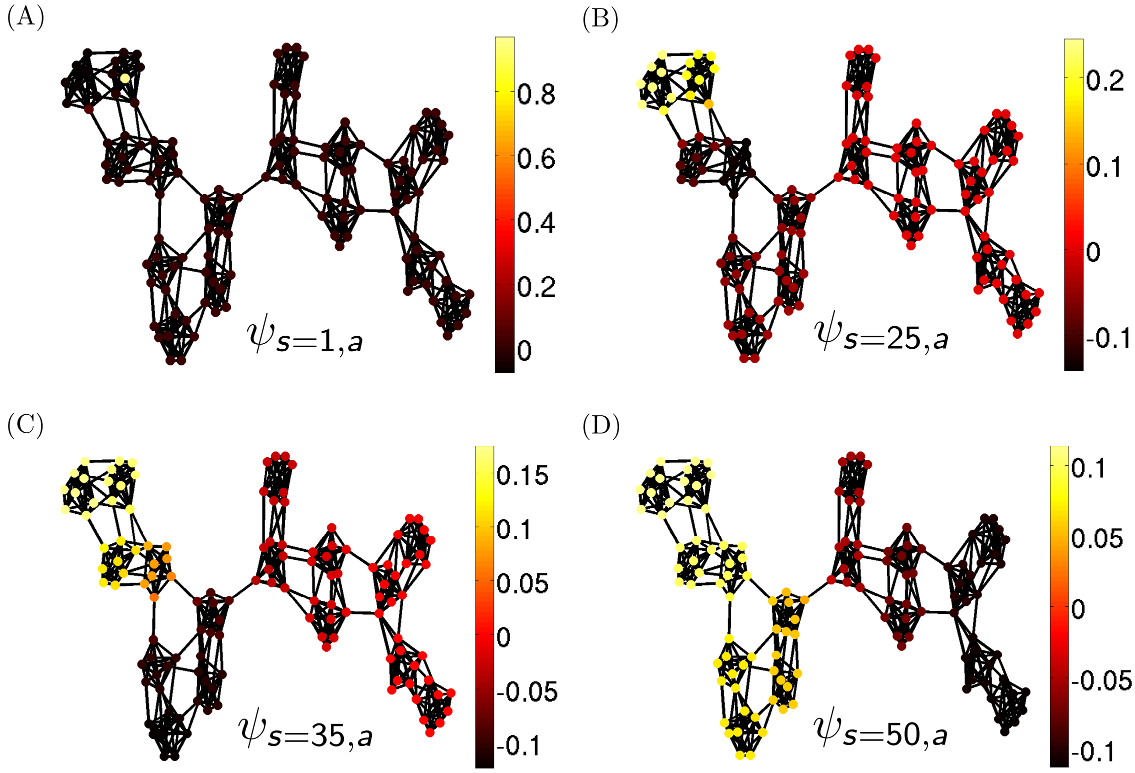

Figure S3. Wavelets on graph. Spectral graph wavelets of the toy graph with 128 nodes (Fig. S1), centered at the yellow reference node x_k visible in (A). We used $N_{scale} = 100$ scales logarithmically distributed in the range $[s_{min}, s_{max}]$. Scale indexes are 1 in (A), 25 in (B), 35 in (C) and 50 in (D). Nodes are colour coded according to the graph wavelet values on each node using the colour maps at the right.

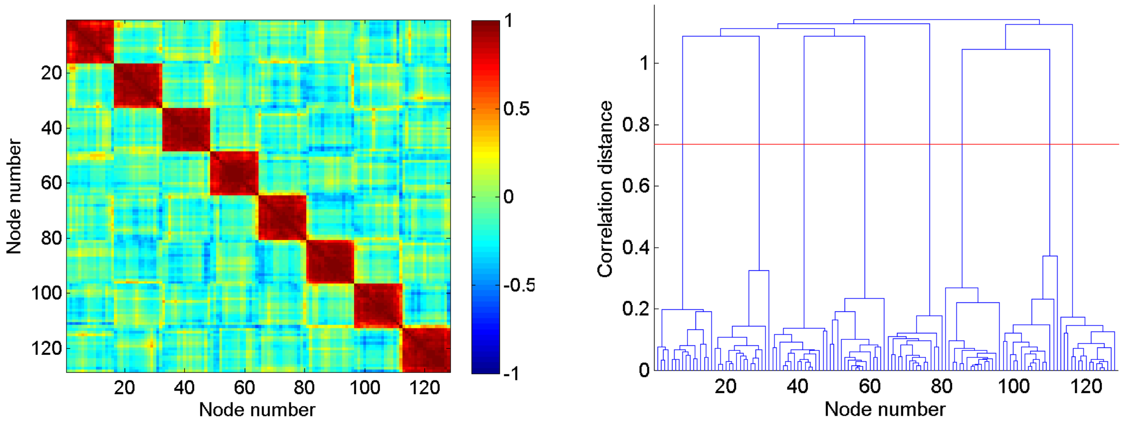

Figure S4. Spectral graph wavelet community mining. Illustration of the usage of spectral graph wavelets on the toy graph with 128 nodes at scale index 25 (Fig. S3B) to delineate graph community at that scale. (Left) 128x128 correlation matrix between the graph wavelets centred on each node at scale index 25. (Right) Dendrogram representation of the hierarchical clustering resulting from the graph wavelet correlation matrix; the red line mark the *best cut* level used to cut the dendrogram and to define the graph communities at the scale of analysis. Nodes where ordered according to the dendrogram representation. 8 communities are delineated, they correspond to the 8 red squares clearly apparent on the correlation matrix and they perfectly recover the 8 groups of 16 nodes at the second level of construction of the hierarchical toy graph (Fig. S1B).

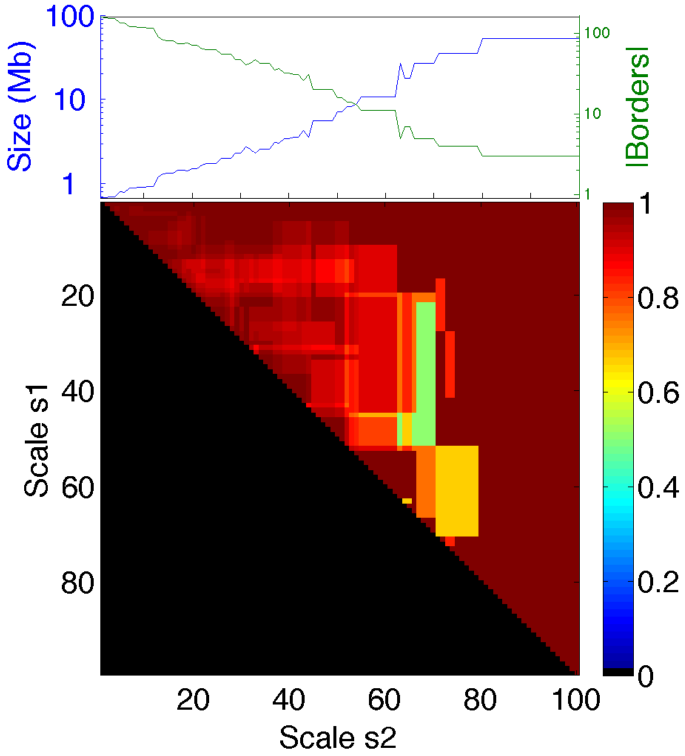

Figure S5. Conservation of community borders across scales. (Bottom) Proportion of borders at a scale $s2 > s1$ that are borders at scale s1, for human chromosome 12 in H1 ES cell line. Note that the smallest proportion of border recovery (~40%) is obtained at a scale $s2$ at which we have 5 borders and only two are recovered. At scale $s1$, the three missing borders being shifted few pixels away from a scale to another. (Top) The average size of communities and the number of borders as a function of s_2.

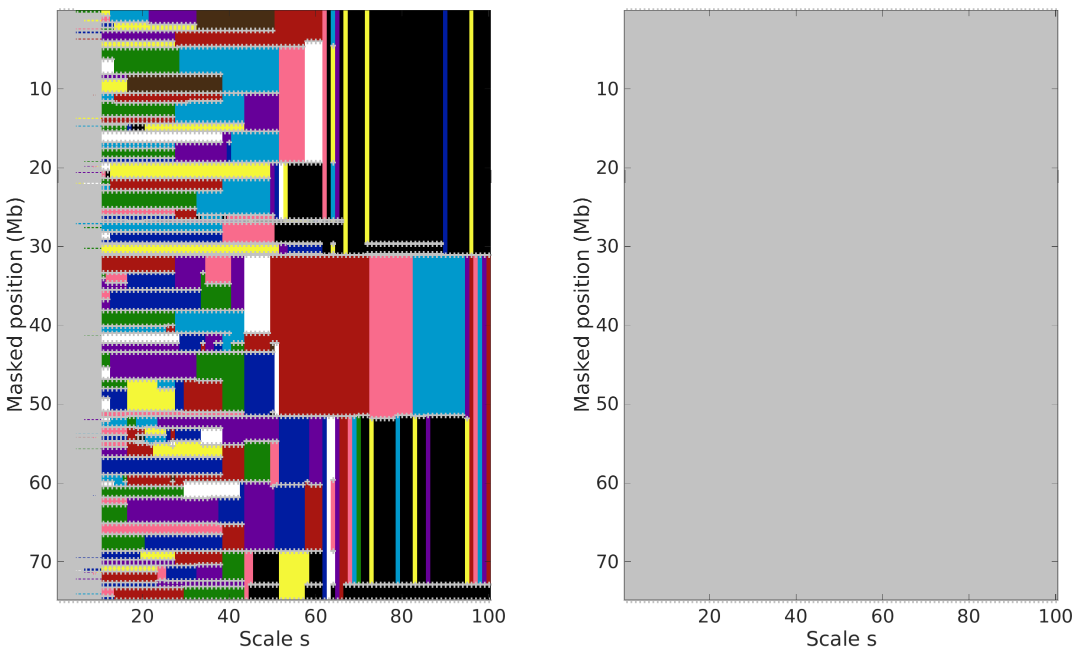

Figure S6. Structural communities during the cell cycle. Same as in Fig. 3 for HeLaS3 cells during G1 (left) and mitosis (right), along the complete masked chromosome 16. Note that mitosis data do not present any structure: at all scales there are no non-trivial communities, each 100 kb locus is a community.

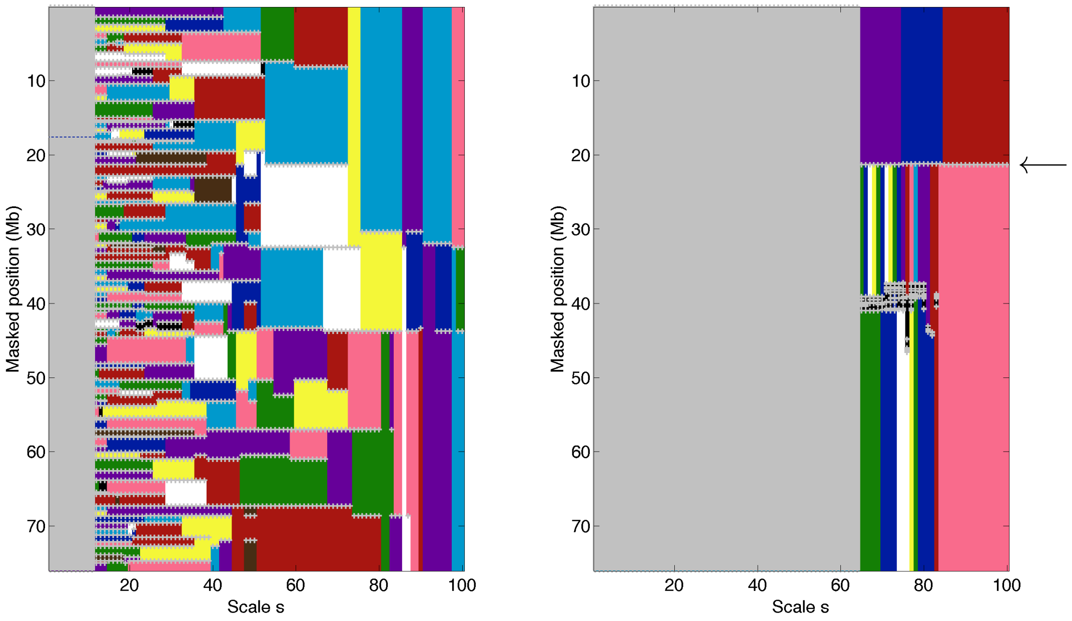

Figure S7. Structural communities during the cell cycle. Same as in Fig. 3 for HeLaS3 cells during G1 (left) and mitosis (right), along the complete masked chromosome 17. The black arrow point to the location of chromosme 17 centromere. Note that the two communities obtained at large scale correspond to the partition of chromosome 17 into its two chromosmome arms.

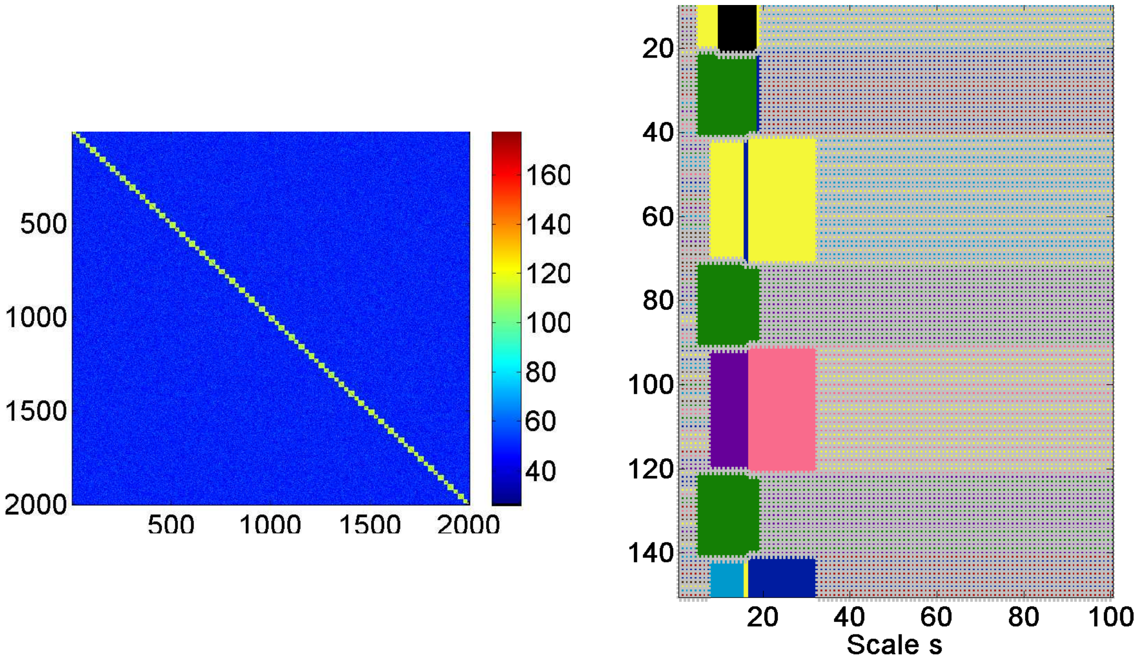

Figure S8. Simulation of a non-hierarchical structural domain organisation. (Left) Model structural interaction matrix for 2000 nodes organised in fully connected interval-communities with no specific organisation at scales larger than the community size: the matrix is built as a series of 40 pairs of domains of size 20 nodes and 30 nodes with internal domain interaction set to 60, with the two first (resp. second) sub-diagonals set to 80 (resp. 70) to assure connectivity and with an additive Poisson noise over all interaction pairs of mean value $\lambda = 50$. (Right) Interval-communities obtained when using the multi-scale community mining algorithm based on graph wavelets on the non-hierarchical interaction network described above and represented like in Fig.3.

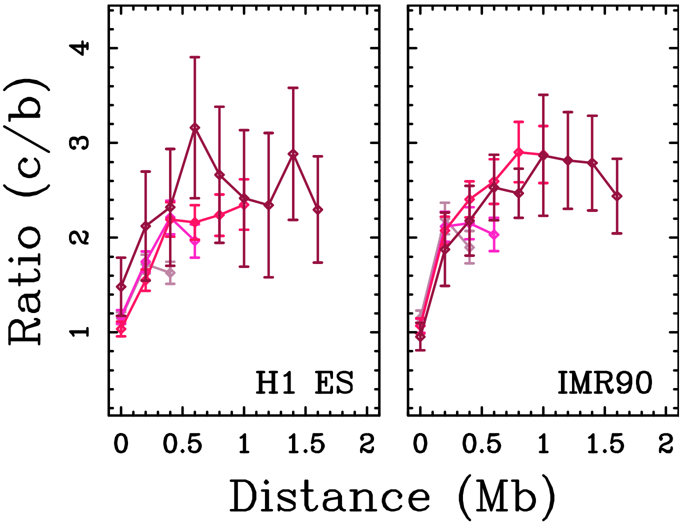

Figure S9. Same as Fig. 4 for the TADs grouped in different size categories: $0.3 \leq L < 0.6$ Mb (light pink), $0.6 \leq L < 1$ Mb (pink), $1 \leq L < 2$ Mb (magenta), $2 \leq L < 3$ Mb (dark pink).

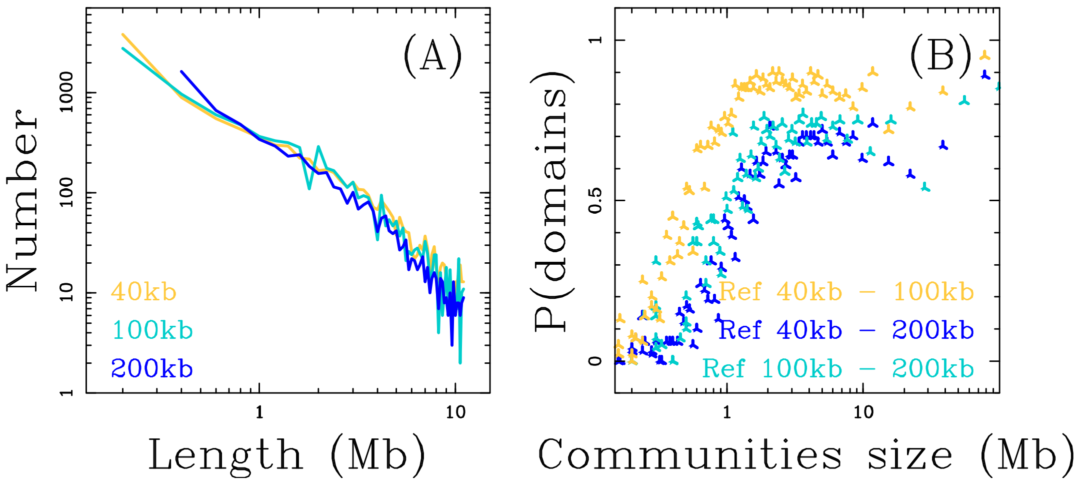

Figure S10. Structural communities detection in the DNA interaction network determined at different binning resolution. (A) Histogram of interval-communities genomic length (l) in a log-log representation for the IMR90 interaction network determined at different binning resolution: 40 kb (yellow), 100 kb (light blue) and 200 kb (dark blue). (B) Proportion of query IMR90 interval-communities determined at a coarser resolution that have a matching reference IMR90 interval-communities determined at a finer resolution: resolution pairs are 200 kb and 100 kb (light blue), 200 kb and 40 kb (dark blue) and 100 kb and 40 kb (yellow). Proportion of interval-community matches is computed over groups of 50 query interval-communities ordered by length (Methods).

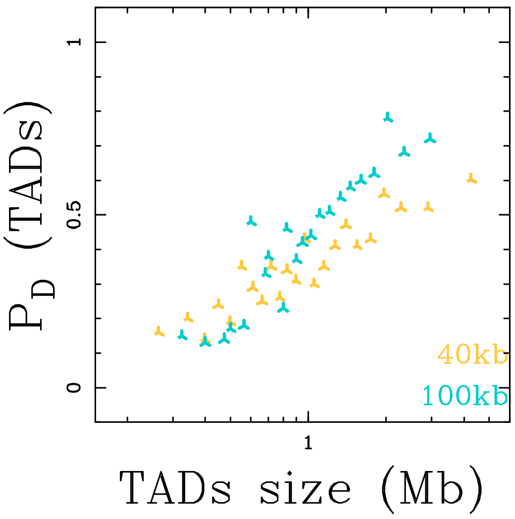

Figure S11. TADs are interval-communities. Proportion of IMR90 TADs that have a match in the interval-community database, as functions of the average TAD size at resolution 40 kb (yellow) and 100 kb (light blue) (Methods).

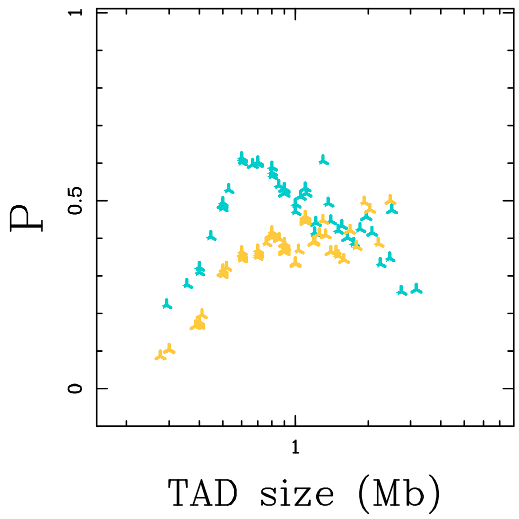

Figure S12. Same as Figure 6 for the comparison of the TAD sets in H1 ES and IMR90. Blue points were obtained with IMR90 interval-communities as the query set and H1 ES interval-communities as the reference set. Yellow points corresponds to the reversed analysis.

Supplementary table

Cell line	Not sequenced	Low interacting	High interacting	Total
IMR90	1792	1100	97	2989
H1 ES	1734	1286	20	3040
K562	1732	565	584	2881
GM06990	1730	990	212	2932
HeLaS3	1836	986	130	2952

Table S1. Masked data. We removed from the original data (28688 100 kb loci over the 22 autosomes) low and high interacting fragments along with fragments corresponding to not sequenced regions of the genome. For each considered Hi-C dataset and for each chromosome, we computed the mean $\bar{c}$ and the standard deviation σ of the total intra-chromosomal interaction count per loci n_i (sum over the Hi-C matrix line). Setting the thresholds to $low = max(0, \bar{c} - 2\sigma)$, and $high = min(0.99L, \bar{c} + 2\sigma)$, where L is the chromosome size (in number of 100 kb pixels), we only retained loci where $n_i \in [low, high]$, removing 10% of the data (6% correspond to unsequenced fragments, $\sim$ 2 to 4% correspond to low interacting fragments and $\sim$ 2% correspond to high interacting fragments).

References

Albert, R. and Barabasi, A.L. (2002) Statistical mechanics of complex networks. *Rev. Mod. Phys.*, **74**, 47.

Boccaletti, S. *et al.* (2006) Complex networks: Structure and dynamics. *Phys. Rep.*, **424**, 175.

Bollobas, B. (1998) *Modern Graph Theory.* Springer, New York, USA.

Bondy, J.A. and Murty, U.S.R. (2008) *Graph Theory.* Springer.

Caldarelli, G. and Vespignani, A., eds. (2007) *Large Scale Structure and Dynamics of Complex Networks.* World Scientific, Singapore.

Chen, H. *et al.* (2015) Functional organization of the human 4D nucleome. *Proc. Natl. Acad. Sci. USA*, **112**, 8002–8007.

Chung, F.R.K. (1997) *Spectral Graph Theory.* American Mathematical Society.

Coifman, R.R. and Maggioni, M. (2006) Diffusion wavelets. *Appl. Comput. Harmon. Anal.*, **21**, 53–94.

Diestel, R. (2010) *Graph Theory.* Springer.

Fiedler, M. (1973) Algebraic connectivity of graphs. *Czechoslovak Mathematical Journal*, **23**, 298–305.

Fortunato, S. (2010) Community detection in graphs. *Phys. Rep.*, **486**, 75–174.

Hammond, D.K. *et al.* (2011) Wavelets on graphs via spectral graph theory. *Appl. Comput. Harmon. Anal.*, **30**, 129–150.

Jansen, M. *et al.* (2009) Multiscale methods for data on graphs and irregular multidimensional situations. *J. Roy. Stat. Soc.: Series B (Statistical Methodology)*, **71**, 97–125.

Mallat, S. (1998) *A Wavelet Tour of Signal Processing.* Academic Press, New York.

Mendes, J.F.F. and Dorogovtsev, S.N. (2003) *Evolution Networks: From Biological Nets to the internet and WWW.* Oxford University Press, Oxford.

Narang, S. and Ortega, A. (2012) Perfect reconstruction two-channel wavelet filter banks for graph structured data. *IEEE Trans. Signal Process.*, **60**, 2786–2799.

Pastor-Satorras, R. and Vespignani, A. (2004) *Evolution and Structure of the Internet: A Statistical Physics Approch.* Cambridge University Press, Cambridge.

Rives, A.W. and Galitski, T. (2003) Modular organization of cellular networks. *Proc. Natl. Acad. Sci. USA*, **100**, 1128–1133.

Sales-Pardo, M. *et al.* (2007) Extracting the hierarchical organization of complex systems. *Proc. Natl. Acad. Sci. USA*, **104**, 15224–15229.

Sandryhaila, A. and Moura, J.M.F. (2012) Discrete signal processing on graphs. *IEEE Trans. Signal Proc.*, **61**, 1644–1656.

Scott, J. (2000) *Social Network Analysis: A Handbook.* Sage Publications, London, 2nd edn.

Shuman, D.I. *et al.* (2013) The emerging field of signal processing on graphs: Extending high-dimensional data analysis to networks and other irregular domains. *IEEE Signal Processing Magazine*, **30**, 83–98.

Spirin, V. and Mirny, L.A. (2003) Protein complexes and functional modules in molecular networks. *Proc. Natl. Acad. Sci. USA*, **100**, 12123–12128.

Tremblay, N. and Borgnat, P. (2014) Graph wavelets for multiscale community mining. *IEEE Trans. Signal Process.*, **62**, 5227–5239.

von Luxburg, U. (2007) A tutorial on spectral clustering. *Stat. Comput.*, **17**, 395–416.

Wasserman, S. and Faust, K. (1994) *Social Network Analysis.* Cambridge University Press, Cambridge.

West, D.B. (1995) *Introduction to Graph Theory.* Prentice Hall, Englewood, Cliffs, NJ.
